# Cytokine Induced Killer cells effectively kill chemo-resistant melanoma cancer stem cells

**DOI:** 10.1186/1479-5876-13-S1-O1

**Published:** 2015-01-15

**Authors:** Loretta Gammaitoni, Lidia Giraudo, Valeria Leuci, Giulia Mesiano, Michela Cangemi, Alessandro Zaccagna, Alberto Pisacane, Susanna Gallo, Fabrizio Carnevale-Schianca, Massimo Aglietta, Dario Sangiolo

**Affiliations:** 1 Candiolo Cancer Institute – IRCCS, Candiolo, Italy; 2 University of Torino, Department of Oncology, Turin, Italy

## Background

Metastatic Melanoma (mMel) is considered refractory to conventional chemotherapies. New molecular targeted approaches, inhibiting mutated forms of the serine-threonine kinase B-RAF, significantly increased the response rate but patients almost invariably relapse and prognosis remains severe [1,2]. Open challenge is the characterization and targeting of cancer stem cells (CSCs), considered responsible for chemoresistance and disease relapse. Adoptive immunotherapy holds great promises for the treatment of mMel and research efforts are ongoing to explore its potential activity against melanoma CSCs (mCSCs). Cytokine Induced Killer (CIK) cells are a subset of *ex vivo* expanded T lymphocytes with mixed CD3^+^CD56^+^ phenotype and endowed with HLA-unrestricted tumor killing activity. We and others recently reported the preclinical activity of CIK cells against several solid tumors including mMel [3].

***Aim*** of our research is to explore the preclinical activity of CIK cells against autologous mCSCs surviving treatments with chemo or molecular targeted therapies.

## Material and methods

We set a preclinical autologous immunotherapy model with primary melanoma cultures and CIK cells generated from patients treated at our Center. To visualize mCSCs we transduced tumor cells with a lentiviral CSC-detector vector encoding enhanced Green Fluorescent Protein (eGFP) under control of the stem-gene oct4 promoter. We treated all mMel cultures with chemotherapy drug fotemustine (IC50 dose). Melanoma cultures harboring BRAF V600E mutations were also treated with BRAF inhibitor (BRAFi) dabrafenib (IC50 dose). We evaluated the presence of residual mCSCs among mMel cells surviving 72h of treatment and explored their susceptibility to immunotherapy with autologous CIK cells.

## Results

We visualized mCSCs within 11 mMel cultures (3/11 BRAF V600E mutated); median value of eGFP+mCSCs was 15% (range 3.5-26.4%). Putative mCSCs displayed a relative resistance to conventional treatments. The presence of eGFP+mCSCs increased of 39% and 25% among melanoma cells that survived exposure to fotemustine and BRAFi respectively, compared to untreated controls (n=11). CIK cells effectively killed autologous mMel and mCSCs. Tumor specific killing, equally involving bulk mMel targets and mCSCs, ranged between 75% and 33% at effector target ratios of 40:1 and 1:1 respectively (n=8).

## Conclusion

We provided first proof of concept that putative mCSCs are relatively resistant to conventional treatments with chemo or molecular targeted therapy and susceptible to immunotherapy killing by autologous CIK cells. These preclinical findings support the hypothesis that mCSC may be responsible for disease relapses and support designing of experimental immunotherapy clinical trials with CIK cells in mMel settings.
